# Using a Communication Model to Collect Measurement Data through Mobile Devices

**DOI:** 10.3390/s120709253

**Published:** 2012-07-05

**Authors:** José Bravo, Vladimir Villarreal, Ramón Hervás, Gabriel Urzaiz

**Affiliations:** 1 MAmI Research Lab, University of Castilla-La Mancha, Ciudad Real 13071, Spain; E-Mail: ramon.hlucas@uclm.es; 2 Systems and Computer Engineering Faculty, Technological University of Panama, Panama; E-Mail: vladimir.villarreal@utp.ac.pa; 3 Division of Engineering and Exact Sciences, University of Anahuac Mayab, Merida 97310, Yucatan, Mexico; E-Mail: gabriel.urzaiz@anahuac.mx

**Keywords:** communication model, data transmission, sensors integration, mobile devices

## Abstract

Wireless systems and services have undergone remarkable development since the first mobile phone system was introduced in the early 1980s. The use of sensors in an Ambient Intelligence approach is a great solution in a medical environment. We define a communication architecture to facilitate the information transfer between all connected devices. This model is based in layers to allow the collection of measurement data to be used in our framework monitoring architecture. An overlay-based solution is built between network elements in order to provide an efficient and highly functional communication platform that allows the connection of a wide variety of devices and technologies, and serves also to perform additional functions such as the possibility to perform some processing in the network that may help to improve overall performance.

## 1. Introduction

The Ambient Intelligence (AmI) concept has emerged to describe interactions between a multitude of network-enabled devices, services, and artifacts. The technology will be nearly invisible, embedded in all kinds of objects and everyday environments such as the home, office, car, and train.

On the other hand, advances in sensors, mobile, and embedded devices have made patient monitoring possible and provided medical treatments and other assistance in health care based on a communication platform that allows the safe transmission of medical data.

**Figure 1.**
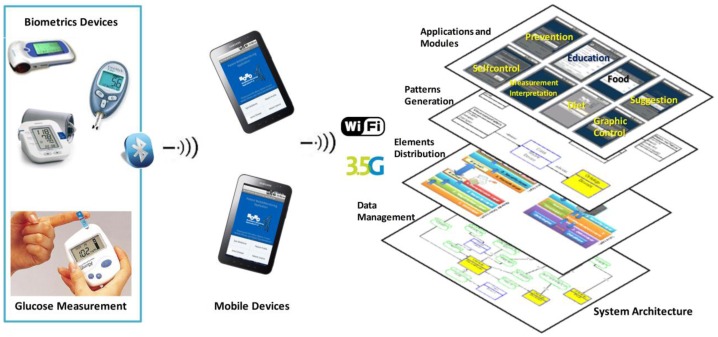
Functionality and elements of the architecture.

We have developed a mobile monitoring architecture that allows patients to have constant control of their vital sign trends as well as direct communication with their doctor. This development is based in a conceptual framework developed by our research lab and this allows the development of each element (user interfaces, modules definition and relation, data processing, and measure acquisition) of the software architecture. Furthermore, we aim to educate these people about their disease; therefore, we are elaborating an educational component meant to allow them to know more about the disease and how to make their daily routines more comfortable. In Figure 1, we show the elements of the software architecture. We have a system architecture that allows the patient mobile monitoring through mobile devices. This software architecture is developed through the definition of specific modules; these modules have been developed with a set of patterns that define the users' interfaces and the module functionality. We have a layer distribution to send and receive data through mobile devices. In this aspect we present this paper the use of a communication model to collect, transmit, and receive data.

We have designed each user's interface with the relation between each control module. We have a software architecture that needs to obtain measurement data from biometric devices to offer a patient constant monitoring. The question is as follows: how does transparent communication help the data transmission between mobile devices? The main objective in this paper is to offer a transparent and timely communication model. In this paper we are defining how each module of the architecture can be connected to collect measurement data. The goal of this work is to develop a communication model that allows the distribution of software architecture elements and to facilitate the development of mobile monitoring applications of any disease. The local application running on the mobile phone no longer needs to know which server to connect with for a specific service, and instead just needs to invoke an object. The communication model is able to provide an efficient and pertinent response to any user requirement, due to the fact that it not only takes into consideration the network conditions but also is aware of semantic relationships. The response may be also enriched as a result of an information fusion process while it travels through the network.

The paper is structured as follows: Section 2 presents a review of some research on the design and development of communication solution applied in AmI. Section 3 presents the developed elements of the software architecture based on a mobile monitoring framework. The use of ontologies and MobiPattern define the relation between the software architecture functionality and the proposed communication model. Section 4 explains each of the layers that define the communication model structure. These layers are distributed in the mobile devices and offer services through the Virtual Quality Network (VQN) core. Section 5 presents the methodology used to integrate all the elements of the architecture. This methodology comprises the defined ontologies, patterns and layers. Next, Section 6 presents the evaluation of the software architecture based on the implemented communication model. Finally, Section 7 presents the conclusions of this paper and future works.

## 2. Background

Many assistive systems have been developed, both for indoor and outdoor environments. Most are focused on a single task such as vital sign monitoring, fall detection, environment customization, or to locate a person. A radio transmitter that acts as an alert beacon when a button is pushed represents a less complex system. Limited effectiveness may be expected from the latter based on the fact that during a heart attack or stroke the patient may not be able to push the panic button. Other systems are based on the use of a public or mobile phone or a pager-like device.

We know other research on assisted living ex0ists; however, that research is based on specific developmental technologies and control devices. In this section we will present our project and discuss the differences between existing architectures and ours.

At Georgia Tech [1,2] the Aware Home project created a home environment that monitors its occupants' whereabouts and activities. The services provided by Aware Home range from enhanced social communication like providing a digital portrait of an elderly person to family members, to memory aids that assist users in resuming interrupted activities by using playbacks of video recordings of past events. Next, in [3] research related to the aspects of home health monitoring using a smartphone was presented. We are developing an architecture that allows the use of different mobile devices with different biometric devices. This architecture generates interfaces based on disease input as a healthcare control.

At the University of Virginia [4] a smart in-home monitoring system has been developed to collect data with the use of a suite of low cost, non-intrusive sensors. The information collected is logged and analyzed in an integrated data management system. The system essentially collects information in a passive manner and does not directly interact with the person being monitored. We collect data from each patient through mobile devices when a patient uses the biometric device. This data is stored on a server allowing for patient control, and to provide suggestions for prevention and progression of diseases.

Intel [5] is focused on improving care in clinical environments, advancing personal health technologies for the home, identifying new care models and work practices, and promoting standards and policies that enable innovation and interoperability across the healthcare ecosystem. Some research development by Intel is focused on Technology Research for Independent Living (TRIL), Everyday Technologies for Alzheimer Care (ETAC) and Aging Services.

At the Center for Future Health (CFH) at the University of Rochester [6] the smart medical home prototype consists of infrared sensors, computers, biosensors, and video cameras. The key services provided are medical advisory ones, providing a real-time conversational interface between the patient and a health care expert, as well as motion and activity monitoring, pathogen detection, skin care, and personal health care recording for consumer-provider decision support.

The ElderCare [7] platform aims at providing a holistic ICT infrastructure for AAL in any home or residence. It is affordable, unobtrusive, easily deployable, usable, accessible, and available. It describes the architecture and components of an AAL-enabling platform, centered around interactive TV (ITV), which combines OSGi middleware, RFID, and NFC in order to ease the day-to-day impact on dependent or semi-dependent elderly people (its main focus), their caretakers, and their relatives.

The communication platform is also one of the most important elements to be considered in modern AmI applications, and it implies enormous challenges from the technological point of view. Some of these challenges are the following: heterogeneity, versatility, high demands, and efficiency. A wide variety of devices should be connected [8]. Other inherent issues (for instance energy consumption [9]) must be also taken into account. Devices may be connected by means of a wide variety of technologies, either with a traditional network infrastructure or through an *ad-hoc* network environment.

Flexibility [10], scalability, and other requirements may also be considered. Highly demanding multimedia applications [11] should be supported with Quality of Service (QoS). Process time is always important, but in some cases may be critical. An efficient use of network resources should also be taken into consideration.

Not many communication platforms exist specifically designed for AmI, and the existing ones are based on traditional communications schemes. Fuentes and Jimenez presented AOPAmI [8]. They described an internal platform structure and how service is provided to support AmI devices, emphasizing the dynamic nature of AmI applications. Their proposal is based on Aspect Oriented Programming (AOP) that provides a solution for the problem of managing the evolution on different levels, and therefore it may be applied to develop an AmI platform. Hermann [12] describes an “*ad-hoc* service grid” (ASG) infrastructure to support interactions between mobile users with remote resources along with their current physical environment. An adaptive, dynamic end decentralized service location algorithm for AmI environments is proposed, which generates a global location pattern that minimizes communication costs without the necessity of a central controller.

**Figure 2.**
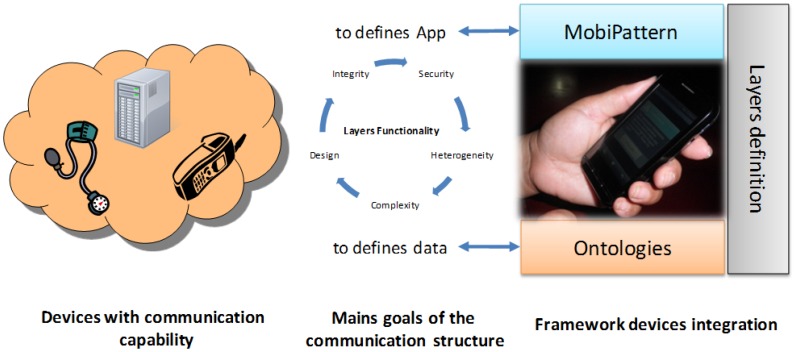
Distribution of mobile devices that interact with the software architecture.

Our proposal is to apply an overlay-based solution between elements in the network (mobile phones, desktops, servers, *etc.*) in order to provide a solid and versatile communications platform. In Figure 2 we explain the elements that make up our architecture and the relation with each layer that will be defined in the next section. We have a devices cloud with communication capability. These communication conditions are defined for the communication model explained in this paper. Each layer has a specific functionality related with each element of the framework architecture. For example, we defined data with the ontologies and define applications with the *MobiPattern*. The relation between ontologies and the *MobiPattern* are defined with the layers distribution.

The communications platform allows for the connection of a wide variety of devices and technologies, and also serves to perform additional functions such as the possibility to perform some processing in the network that may help to improve overall performance. The measuring device will send the data collected from its sensor to the local device (mobile phone) via Bluetooth. If the mobile phone does not have this communication technology, then the patient writes through the device keyboard. However, most of the other devices (servers, other mobile phones, *etc.*) in the network may be connected by means of different technologies.

In order to improve the communication between patients and doctors, this architecture provides, additionally, continuous patient monitoring and supports an automatic patient architecture for the individual profiles, self-control, and education modules for patient's condition. The communication structure defines the communication platform to enable functionality of the measuring devices for each kind of condition, the trend management and the doctor and patient modules.

## 3. Elements of the Software Architecture Based in Mobile Monitoring Framework

Patient monitoring represents one of the key elements in the progress and control of his illness. This monitoring should offer the patient and doctor constant data regarding the disease's status (vital signs, pulse, glucose levels, *etc.*) so that the doctor can readjust the initial treatments and prescriptions accordingly.

This is our motivation in developing the framework architecture for patient monitoring via mobile phone. The mobile phone represents the major technology advance used, which we use for more than 60% of our daily activities.

The development of our framework includes the design of complete and integrated ontologies for knowledge generation and the design of pattern-based software engineering to design all the architecture. Additionally we design a distributed structure communication system to allow the transmission and processing of vital signs between mobile phones and biometric devices. We introduce both elements continually. In this paper we center on the communication structure to define the vital sign transmission model.

### 3.1. Using Ontologies in the Development of the Communication Model

**Figure 3.**
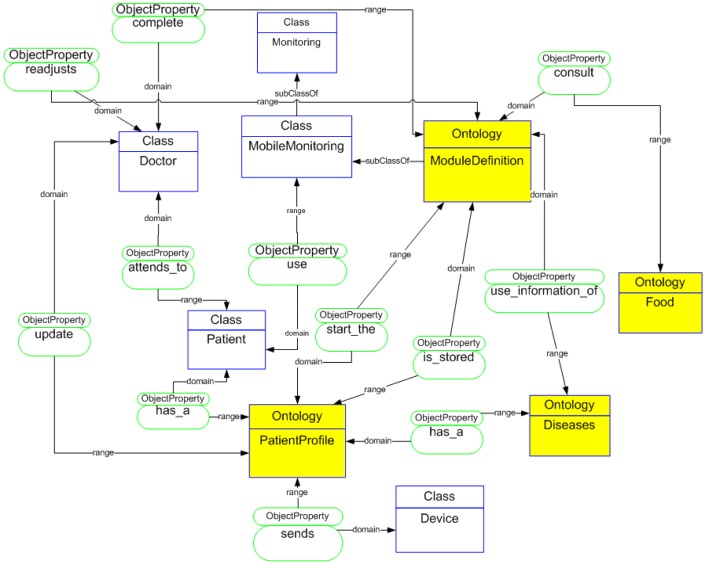
Ontology distribution used for the communication model.

The use of ontologies in AmI brings several benefits and additional functionalities. For data management we develop a complete ontology to define all elements of the architecture (Figure 3). We have previously explored the use of ontologies for runtime adaptation of applications and personalization to particular users' needs [14]. More particularly, the ontological definition has been based on the proposals of Hervás [15] that include a user-centered classification comprising the user, environment, devices, and services. We have enhanced that ontological model with information about the diseases, biometrical devices, and module generation. This ontological distribution allows the development of layers for the distribution and processing of measurement data. This distribution data is being designed in the layers structure for the design of the communication model. In addition to using ontologies developed to understand the environment in which we have worked, they are used for implicit or explicit information. This information is related to the parameters of medical control of the patient. Ontologies will serve in the future to make the developed applications interoperable with other external applications and to implement inference engines that equip themselves with greater intelligence monitoring applications.

The ontological model is formed by three key elements: *PatientProfile*, *ModuleDefinition*, and *CommunicationStructure*. The *PatientProfile* defines each patient's data; *ModuleDefinition* elements generated according to each patient's profile and *ComunicationStructure* define a communication between mobile devices and the framework. For a better understanding of each of the elements in the architecture an ontological classification of the patient's profile is presented as well as of the modules' definition. This allows us to go into each of the functionalities that make it up in depth.

### 3.2. Designing Patterns to Connect All the Elements of the Communication Model

Some researchers define a software pattern structure to develop applications and data interchange [16,17]. The system architecture we have developed allows the construction or generation of interactive applications to be embedded in the mobile device. For the creation and integration of these modules into the mobile devices, we have defined and developed a set of patterns called *MobiPattern*. In any *MobiPattern* we have to consider that all the interpretations are generated after the measurement. *MobiPatterns* define the schema of each screen of the final application and the functional structure. We defined patterns for the use in any application that allows patient monitoring. We organized all measurements obtained by biometric device reads in five levels of ranges: alert, low, acceptable, ideal, and high. This classification allows the generation of an exact medical control for the patient. For this work, we defined layers for each device (mobile phone, biometric devices, and server). Each layer allows the integrated, complex and extracted data to be obtained and allows for processing between devices with capability to transfer data.

## 4. A Layer Distribution for the Definition of the Communication Model

In some cases more orderly and standardized interpretation aspects of communication and data transmission has been studying in different areas in aspect about layers distribution.

Layer architecture allowing for greater interoperability of all architecture and facilitating standardization development has been implemented in different areas [18,19]. In our research, we have defined and developed the layer architecture of our framework. We developed a communication model that is made up of three distributed layers in all devices that have the mobile monitoring software architecture installed.

**Figure 4.**
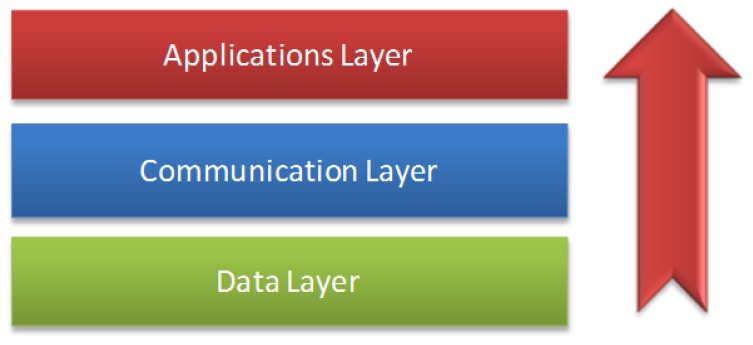
The three layers of the model.

This architecture is distributed through the three main elements: patient's mobile phone, doctor's personal computer, and a central server, as shown in Figure 4. The framework server layers define the design layer, which establishes the communication with elements of the ontologies and the module's generation.

A communication layer parameter established the data transmission/reception to/from the mobile phone. In addition, a security layer provides *encryption* for data transmission. After embedding the application on the patient mobile phone, we defined the layers that comprise it. At the top we have the applications layer, which contains all modules generated by the framework corresponding to the elements of the final application. The next layer defines the security levels to prepare the receiving layer in the collection of data from the biometrics devices. The link layer that established the parameter to connect with the mobile phone makes up the biometric devices. Also, the negotiation layer allows the selection of the type of connection (Bluetooth, NFC, *etc.*). Finally, the security layer is defined to establish the type of data *encryption*, and the transmission layer to allow the transfer of each measurement.

### 4.1. Data Layer Functionality

**Figure 5.**
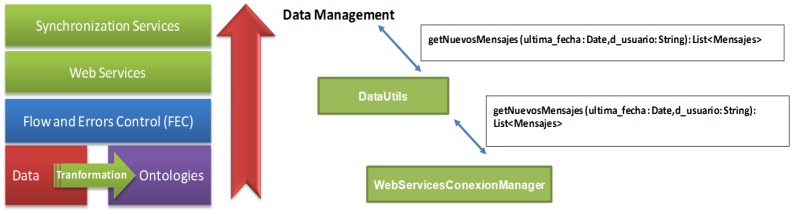
Elements of the data layer (**left**) and the flow data that define this layer (**right**).

The data layer specifies the elements to be taken into account at the time of generating applications. For this, all necessary components have been defined through ontologies, which establish relations between each module and define the reading and writing parameters as needed for each case. These ontologies are defined in the database that stores all the information related to all data obtained from a patient profile (*measurement readings*) and the data associated with a disease and the operation for each of the modules generated for this type of disease on the one hand. In Figure 5, we show the distribution of data layer. The functionalities of this layer are as follows:

- Start the identification of roles and access to the application.
- Reply synchronization requested by the mobile application.
- Establish aspects of control flow and access (FEC) of each module of the final application.
- Establish the relationships between the elements of each defined ontology.
- Providing services to the inference engine upon request.
- Processing the data requested in the query generated from the mobile device.
- Offer Web Services for each link of mobile devices.
- Update the database according to measurements obtained from the mobile device.
- Manage and coordinate the communication between the server and the mobile device through defined Web Services.

### 4.2. Communication Layer's Functionality

**Figure 6.**
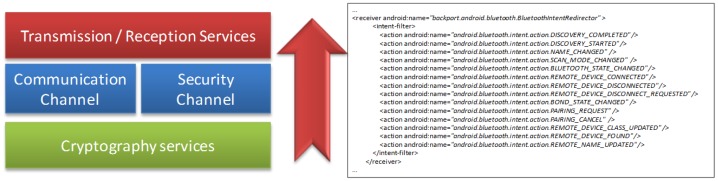
Elements of the communication layer (**left**) and basic code to communication process generation (**right**).

The communication layer establishes the parameters of communication between the primary server and mobile devices of each patient, keeping the information updated on an ongoing basis. This layer also facilitates the updating of previously generated applications, depending on the needs with regard to the patient's evolution, which is so that adjustments can be made in the event that complicate a disease or a patient improves with treatment. Figure 6 shows the elements of communication of the server layer, summarized in the functions that are part of the communication layer:

- Enable the channel of communication between the server and the mobile device.
- Provide security services for the transmission of data.
- Offer mobile device access to the services offered by the data layer.
- Receiving encrypted requests for passing on to the security layer.
- Enable *n* numbers of channels that allow the simultaneous access of several mobile devices.

#### 4.2.1. Implementation of the Communication Platform

**Figure 7.**
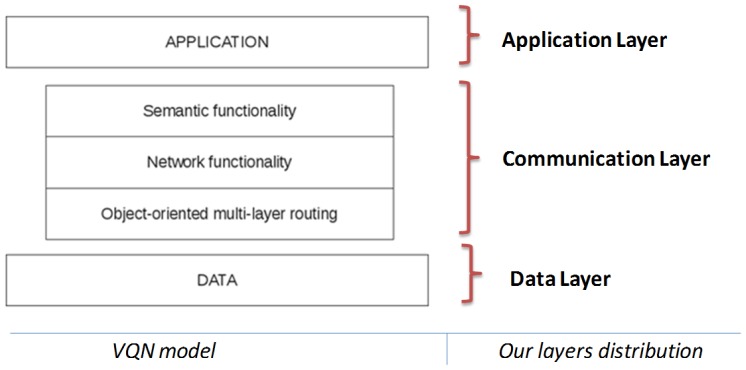
Major components of the VQN model related with our layers distribution.

Implementation of the communication layer is based on the Virtual Quality-of-service Network (VQN) model [13], a semantic overlay network implemented as a distributed application by means of an object oriented middleware for distributed systems. VQN was designed to support a heterogeneous environment. This means that a wide variety of devices (such as mobile phones, PDAs, portable computers, servers, *etc.*) could be connected transparently. The overlay network is divided in three sub-layers. This division helps to maintain an order through the enhancement process, and gives independence between the functions that each one of the sub-layers performs. *VQN* includes the following three major components: object-oriented, multi-layer routing; network functionality; and semantic functionality (Figure 7).

The object-oriented, multi-layer routing [20] function is used to provide an end-to-end transport service that is independent of any network technology or protocol. The implementation of the object-oriented, multi-layer routing as a coupling element between the overlay and the underlying network provides advantages in performance and functionality when compared to conventional TCP/IP mechanisms. The definition and development of layers in the framework allows for the design of user interfaces.

The network and semantic functionality layers offer the possibility for the participating nodes to perform additional functions depending on their characteristics and circumstances. This additional functionality is not restricted to just network-type operations but it could also be extended to address additional requirements, even at the semantic level.

A simple application is presented here in order to illustrate the functionality provided by VQN. Consider a mobile phone that requires some process done in a remote location, probably by means of a heterogeneous connection dealing with different technologies, such as Bluetooth, Ethernet, WiFi, GPRS, TCP/IP, *etc.*

Consider the case of a patient who is constantly being monitored for vital signs such as temperature, blood pressure, physical activity, and insulin level by means of the appropriate measurement devices, all of them probably provided with a Bluetooth connection to connect to the mobile phone. All these variables and periodic measurements are sent to the mobile phone, which has an application that receives and stores all this information, and based on a diagnosis and if possible it gives directions to the patient locally. In some cases there may be necessary to call a doctor or an ambulance, or ask a pharmacist about a specific medicine, *etc.*

**Figure 8.**
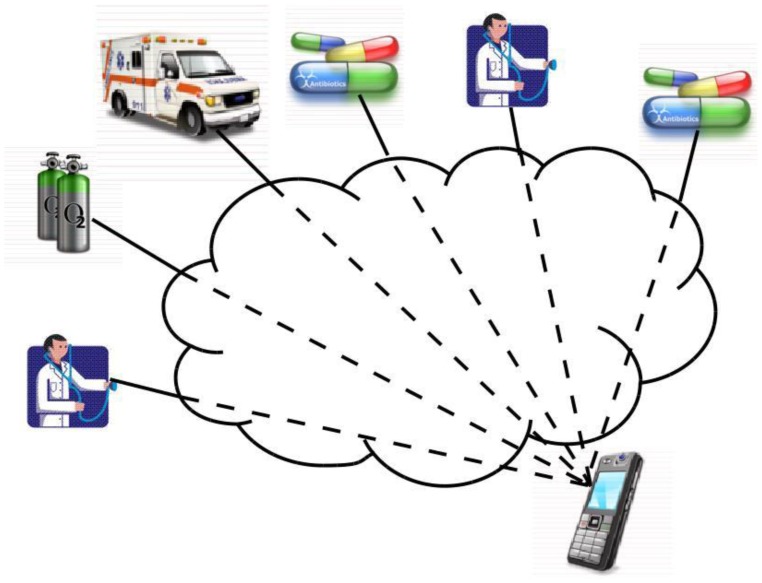
Server process requirements in a traditional solution.

A traditional solution to this situation (Figure 8) would include some servers in the cloud to provide the required process function. The application running in the mobile phone needs to know about every service in the network and how to reach each one of them. Somehow it decides which specific server to connect with and sends to it the request. Then the server performs the required process and sends the answer back to the application at the mobile phone.

**Figure 9.**
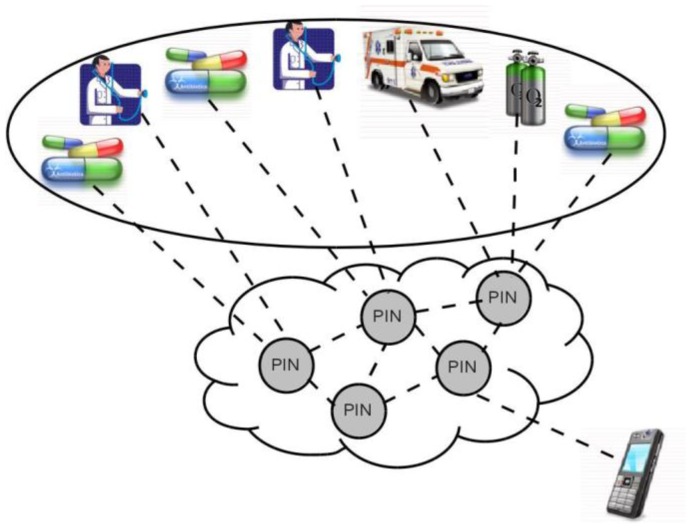
Alternate solution with PIN functionality.

Once the VQN platform is established between devices (Figure 9), the local application running on the mobile phone no longer needs to know which server to connect with for a specific service, and instead just needs to invoke an object. For instance, the application on the mobile phone just needs to invoke an ambulance object, and the network is in charge to find the ones that are available at any given time, and even to decide which is the nearest, or the cheapest, or the most appropriate based on semantic similarity, *etc.* and establish the connection accordingly.

VQN also opens a wide variety of network services that could be easily implemented such as the possibility to perform some Process-In-Network (PIN) [21], as an example of the additional functionality that could be provided. PIN should be understood as the possibility of performing some processing as information passes through the network. The processing is done directly in the network nodes that are found between the origin and destination, taking advantage of waiting times in queues of routers, idle processing capacity in the intermediate nodes, and the information itself. This means that some process (or even the whole process) may be performed before the requirement arrives to the server, and therefore a lower requirement of time and process at the destination end nodes. Information may also be simplified and even semantically enriched as it passes through the network.

### 4.3. Application Layer's Functionality

In the application layer are the applications generated by the framework for monitoring and self-control of the patient. These applications are based on ontologies specific to each disease and the individual profile of each patient. It also boasts an inference engine, which is updated every time a reading is generated from the biometric device. This is responsible for requesting changes to future applications previously generated for the mobile device.

The application layer provides the user with a graphical interface that allows maintaining a constant and continuous monitoring of the patient including the medical parameters or vital signs obtained from biometric devices, as well as control the monitoring process.

In addition, the application layer makes reference to the organization and distribution of all the modules that make up the application layer. This distribution allows for the correct worksite of each of the modules, providing specific functionality within the application. Also, we have another function of this layer:

- Distribute the elements of each module.
- Establish relationships between each element.
- Offer services of internal connection in the application.
- Locate in levels, according to the degree of interaction, each of the elements of the application.

**Figure 10.**
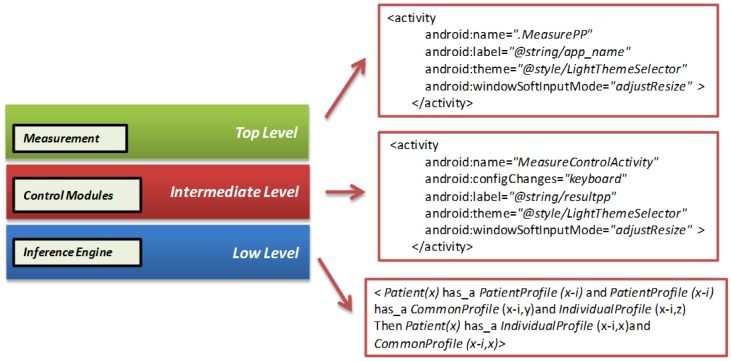
Elements of the application layer (**left**) and some examples implemented on each level.

Figure 10 shows the distribution of each module depending on the level in which it has been initially placed. This distribution refers to the location in memory of each sub-module. This layer is composed of the following levels:

- **Top level**: the actions module, which is responsible for the measurements of the patient, is located at this level.
- **Intermediate level**: all the other modules of the application are located on this level; profile, recommendations, prevention, alarms, diets, *etc.* are located within this level.
- **Low level**: the inference engine, responsible for the interpretation of the information updated, based on past situations is located at this level.

## 5. Integrating Applications in Mobile Devices

Two specific areas comprise the architecture developed in this paper. The first area corresponds to development of the application for the doctor. The second area corresponds to the application for the patient. The development of the software architecture follows the methodology we designed. This methodology has the following steps:

- Selection of the module to implement: the functional structure of each module that will be part of the final application is designed. Each module has a specific functionality, based on an overall associated design.
- Definition of design patterns: defining the physical structures of each pattern associated with each module that will run. Specifies the visual of each of the modules design.
- Definition of functional patterns: defines the roles and relationships of each of the modules of the application.
- Ontological relationship of each module: specifies the ontologies that are involved or are used by each module as well as the relationship between other elements of architecture.
- Determination of the layers where comes the module: defines the functional layer of each module, relating it to the model layer, defined by the framework.
- Determination of the inter-modular relationship: defines the relationship between each of the developed modules, allowing interoperability between each of them.
- Integration of all elements: for obtaining the prototype to evaluate. In this step, an initial prototype is obtained.
- Evaluation of the prototype: allows the evaluation of the function and visual design of the application generated, providing feedback for the improvement of the architecture. Redesign of the elements for the generation of a new prototype - here is where the functionality of the retrieved prototype is discussed for its redesign according to the initial steps.

**Figure 11.**
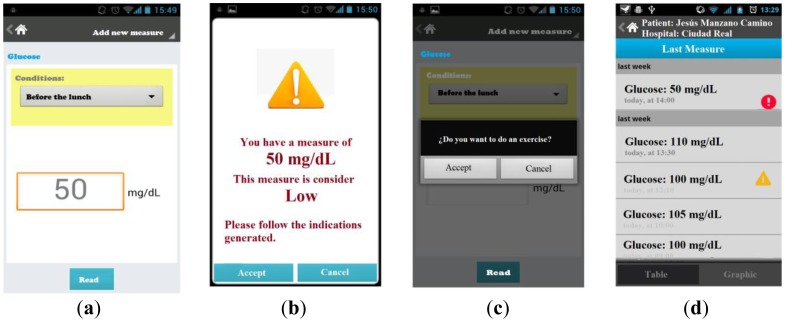
Generation of the final application based in our framework.

The application for the doctor is embedded in the mobile device; from there it controls all patients' records, organized by each patient's profile, the history of the patient's measured data was controlled, and specifications of the disease(s) were recorded. Some screens of the mobile monitoring application based in the communication platform are shown in Figure 11.

The communication and model applications are developed in the Android Operating System 2.3 with remote connectivity through MySQL. The database and all the specifications of the framework are distributed on the main server; the doctor and the patient have access to these data every time one uses the application for each of them.

The patient has a screen with a menu that allows him to obtain access to all functions of the application. The patient can update information in the profile; if the patient needs to update this information, they just need to change specific data about the specific profile.

Each collects measure data that is associated with a specific *patient id*, which allows the update to the server for the doctor to interpret the measurements of each patient without confusing them with others. The reading of the measurements can be manual, where the patient enters through the keyboard obtained measurement and automatic reading of the measure obtained directly from the biometric device via Bluetooth technology.

The patient can select the type of disease that he wants to graph, from data captured from the biometric device. Our architecture can display graphs of different diseases. Our first assessments captured measures of diabetes, blood pressure, and temperature. These graphics or table of measures depend on the type of disease(s) the patient has (in some cases may be more than one) and the changes obtained during a period of time.

The patient can see his latest measurement obtained either through a chart showing the extent and period of time that it was taken, or through a table that stores information about the day and time, as numerical value, analyzed range from the application (low, normal, high).

## 6. Evaluations, Simulations, and Proof of Our Proposal

We present two types of evaluation. In the first type we present the evaluation of the functionality of the developed software architecture. Aspects about content, design, and usability of the software architecture are evaluated. In the second type we present the communication model evaluation. This evaluation first included a proof of concept to demonstrate feasibility of the communication platform when the software architecture is running, and afterwards a simulation test to illustrate the network enhancement that could be provided by the implementation of PIN functionality specifically in regards to the PIN functionality of network enrichment in communication layer of the software architecture.

### 6.1. Evaluating the Software Architecture in the Final Users

**Figure 12.**
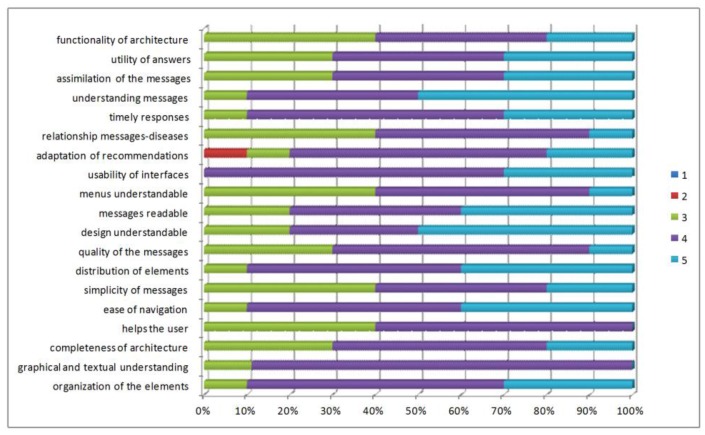
Results of the quality aspect evaluated for the final users.

We present an evaluation of the functionality of the developed software architecture. Patients with diabetes and high blood pressure, needing to provide follow-up to the disease, have evaluated these domains. We assessed aspects of content, design, and usefulness of architecture in the questionnaire provided to the patient (Figure 12).

To evaluate the content criteria, the patients responded to questions related to the organization of the content, the presence of help during the use of the application, ease of interpretation, ease of identification of the elements, and degree of value of the submitted content. For the design criteria, they responded to questions about the distribution of visual elements, displays, interpretation, and identification of menu specifications. For the usefulness criterion, they responded to questions about the degree of usefulness of the generated recommendations, messages of prevention and education, and degree of satisfaction with the information.

Initial aspects of the evaluation:

- **Evaluation technique**: A questionnaire was applied where participants respond to specific questions about how they used the application.
- **Aspects of quality to assess**: We assessed the aspects of content, design, and usefulness of the application to end users.
- **Context to assess**: The patient used the application to monitor vital signs associated with his illness for a certain time, reviewed the medical control activities (recommendations, messages and prevention, education, and self-control), and added physical activities.
- **Population to assess**: The assessment has been applied to ten people (six men and four women). The population has been formed by two doctor candidates, one doctor, one university undergraduate and six persons unrelated to the university between the ages of 25 to 60. Users associated with the university are related to technology while non-university users have little knowledge of technology.
- **Time to assess**: The time that the patient will take using the application in the defined context will be 45 min. The patient has 15 min to respond to the evaluation questionnaire.
- **Scale to use**: A Likert Scale from 1 to 5 has been established to evaluate each question: 1 being the lowest evaluation for a question (very much in disagreement) and 5 being the highest evaluation (very much in agreement).

The majority of participants considered that the generated application has good content, evaluating between 4 and 5 the aspects such as ease of navigation, understanding of the elements that make up the application, clarity in the information shown, among others.

The design criteria has been the more highly valued feature by the participants, which is due to the fact that they have been careful at the time of establishing the appropriate parameters for the design and functionality of interfaces. Weights between 4 and 5 according to the total number of participants have been obtained. The majority of patients, on the other hand, feel that the application offers an interface or screens that provide good explanatory understanding at all times about what the application is doing.

In general, the results of the evaluation carried out in this first approach have been quite satisfactory. The aspects that have a lower evaluation have been those of utility, which may be due to the notion that patients are used to making annotations in a booklet, and on other occasions do not make annotations, so do not perceive the difference when using the application evaluated.

### 6.2. Evaluating the Communication Model after the Software Architecture Was Used for the Patient

#### 6.2.1. Proof of Concept of the Software Architecture

A proof of concept was implemented by means of a software prototype to demonstrate the feasibility of the communication platform in the monitoring software architecture. The software prototype was built with full VQN and PIN functionality. The implementation of the VQN model included the following features: QoS-aware, multihop, forecasts, and fuzzy logic. The implementation of PIN included the following functionalities: pre-processing, simplification of information, and information enrichment.

The prototype was written in the C++ programming language, complemented as a distributed application by means of an object oriented middleware for distributed applications (*i.e.*, ZeroC ICE). The development was done on an IBM-compatible PC with Intel P4 @2.24GHz processor, MS Windows XP Professional operating system SP3, MS Visual Studio 2005 SP1, and ZeroC ICE v3.3.0. The software prototype was also migrated to Debian GNU/Linux 6.0 (squeeze) with ZeroC ICE 3.3.1-12.

**Figure 13.**
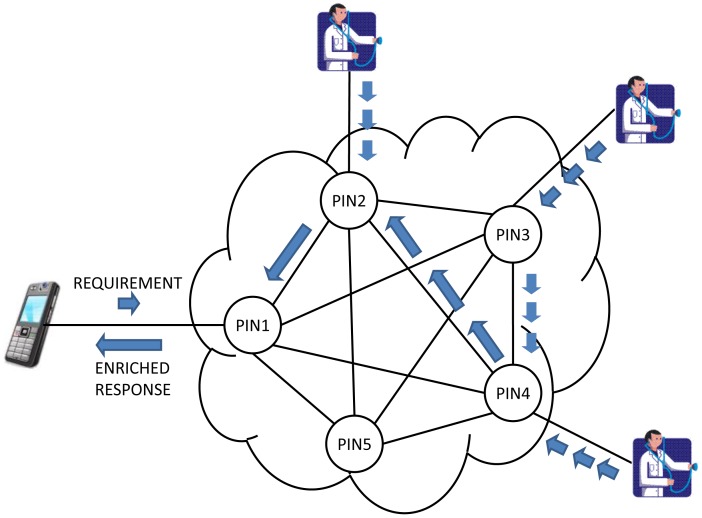
Proof of concept test scenario.

A 5-node, full-mesh topology was considered for the test scenario (Figure 13). Connectivity between nodes was based on ZeroC ICE interfaces, allowing the connection of nodes by means of input buffers.

The proof of concept included two specific experiments. First, we tried to prove that the network could perform the function of choosing from a table of semantic features of the nodes, the destination node best suited for a given requirement. Secondly, we implemented the possibility that for a given requirement it could have an enhanced response formed from the replies of all semantically related nodes. It is noteworthy that in both experiments all the described functionality was programmed into each of the network nodes, performing its function without support in the applications.

#### 6.2.2. Software Architecture Simulation Test

We also conducted an experiment on a network simulator in order to evaluate the PIN functionality of network enrichment into the monitoring software architecture. The objective was to measure the information enrichment that could be obtained by using no more than just the waiting times in the router queues and the idle resources in the network. In other words, the objective was to measure the information enrichment that could be obtained at zero-cost PIN. This evaluation is the result of the previous user evaluation, we obtain the times and the numbers of packages transfer in the devices that interact in the mobile monitoring process.

We firstly defined an Information Enrichment index (IE index) that could be understood as the number of pertinent semantic-related nodes, which the network takes into consideration when building an answer to a specific requirement. The IE index is expressed as a percentage, and it is calculated according to the content of information in the original answer to a user's request. A benchmark of 100% is firstly considered meaning that the original message provided by one destination node has in itself the total content of needed information. A value less than 100% would mean that content has been lost; and a value greater than 100% means that it has enriched the content of information, due to the fact that more than one node provided a relevant answer to the original requirement. All this information was used to build and provide an enhanced answer to the original request.

For instance, suppose that a patient wants to contact a doctor and sends his requirement, and the network provides an answer based on the requirement, and built over what three doctor-type nodes that answer, that are available, and that are semantically related to that specific requirement. In this case, the Index of information enrichment would be 300%. It takes a certain amount of time to build an answer to a requirement, and it is very important to compare it to the average time in the router queues, trying to determine which part of that process could be done at zero-cost. This is by using just the idle time in the network.

The experiment was executed in an IBM-compatible PC with an Intel Atom @1.6 GHz processor, running Debian GNU/Linux OS with g++ compiler and OMNeT++ v4.1. Three different network topologies were used (10-node partial mesh, 5-node partial mesh, and fish) with approximately 40% of their semantic-related nodes. This means that all those semantic-related nodes could provide a valid answer to the original request.

The experiment was useful to measure not only the IE index, but also the time that the network needed to deliver all pertinent answers from the source semantic-related nodes to the destination node that originated the requirement. This delivery time included not only the transmission time but also the processing time used at the intermediate nodes needed to fuse arriving information with the existing one in the node.

Most packets (or even all of them) would eventually arrive at their destination, but not all of them do it in less than the average time in the router queues. In other words, not all packets would arrive to its destination by using just the waiting times in the router queues.

In order to calculate the final numbers for the experiment, only the packets that arrived to the destination node in less than the opportunity for PIN numbers were considered. The IE index at zero-cost PIN numbers are calculated as the average of the IE indexes of those packets that arrived within the average queuing time limits. Final results are summarized in Table 1.

It could be noticed that in all cases information could be enriched with zero-cost PIN. This means that in all cases it was possible to build an answer with enriched information (by means of the use of more than just one answering node) with no extra cost in terms of network resources.

## 6. Conclusions and Future Works

Our main goal with this project is to promote the easy day-by-day life of people with a chronic condition. This architecture provides continuous patient monitoring to improve the communication between patients and doctors allowing the generation of an automatic architecture for the individual patients' profiles of each patient, self-control, and education modules for their chronic diseases. This has been developed for the mobile monitoring of patients via biometric devices and a mobile phone.

An ontological architecture classification has been created. These elements are the patient profiles, where the personal details of the patient are specified, and the definition of the modules for the mobile phone as well as for the doctor. Diet definition, medical treatments, care activities, and patient profile are some of the aspects that have been modeled in the ontologies, and that allow the framework an accurate interpretation to generate the right applications.

An overlay-based solution is used to build a solid and versatile communication platform between network elements, and to provide additional functionality such as PIN. We are confident that good communication will help doctors and patients make better decisions in different situations and conditions.

Future research areas are as follows: assess the architecture in other medical environments that can be monitored to know the effectiveness against different diseases; implement architecture in other environments or platforms for development to be launched in the largest number of mobile devices; a constant assessment of aspects of control of each of the diseases, in such a way that they can adjust to changes in the scientific world; add new biometric devices to obtain vital signs; and develop a module's connectivity to a database of medical centers to add more information about the architecture. Additionally, we are working on achieving a more intelligent behavior of our applications by means of rules based on Semantic Web principles. This field has been explored in previous works applied to different scenarios [22].

## Figures and Tables

**Table 1.** Information Enhancement index at zero-cost PIN.

**Total Number of Nodes in the Network**	**Number of Semantic-Related Nodes**	**Average Queuing Time (Seconds Per Package)**	**Average IE Index at Zero-Cost PIN (Percentage)**
10 (partial mesh)	4	0.06423	278.44%
5 (partial mesh)	2	0.09319	163.03%
7 (fish)	3	0.16613	181.54%
